# Underwater endoscopic submucosal dissection effectively treats circumferential early-stage esophageal cancer recurring at the scar left by a prior endoscopic submucosal dissection

**DOI:** 10.1055/a-2573-7477

**Published:** 2025-04-15

**Authors:** Ping Yao, Liansong Ye, Zhongshang Sun, Xiaozhong Yang, Yifei Pan, Feng Pan

**Affiliations:** 1 91596 Department of Gastroenterology, The Affiliated Huai’an No. 1 People’s Hospital, Nanjing Medical University, Huai’an, China; 2 34753 Department of Gastroenterology, West China Hospital, Sichuan University, Chengdu, China; 3 12461 Nanjing Medical University, Nanjing, China

A 61-year-old female patient came to our hospital for endoscopic resection of early circumferential esophageal cancer (24–35 cm from the incisors; Fig. 1 **a** ). Five years ago, she had undergone endoscopic submucosal dissection (ESD) to remove semi-circumferential early esophageal cancer (27–32 cm from the incisors). Under endoscopy, scar formation was observed at the site 29–31 cm from the incisors in the lesion ( Fig. 1 **b** ). We performed the wide-tunnel single-line clip traction ESD that we had developed previously for the patient 1 . However, due to severe submucosal fibrosis at the previous ESD site, clip traction could not well expose the submucosa and the cutting line ( Fig. 2 **a** ). Therefore, we used underwater ESD to dissect the lesion ( Video 1 ). First, water was injected into the esophageal lumen. Under the action of the buoyancy of water, the local mucosal layer and submucosa were kept away from the deep muscular layer, and the cutting line in the submucosa was fully exposed ( Fig. 2 **b** ; 2 3 ). The subsequent submucosal dissection process went smoothly ( Fig. 2 **c** ), and no adverse events such as muscular layer injury occurred ( Fig. 2 **d** ). The entire lesion was resected en bloc ( Fig. 3 **a, b** ), which took 120 minutes. The postoperative pathological results showed moderately differentiated squamous cell carcinoma, invading the muscularis mucosa, with both the horizontal and vertical resection margins being negative. To our knowledge, this case is the first report on the successful treatment of recurrent circumferential early esophageal cancer at the scar formation site after previous ESD by underwater ESD, suggesting that underwater ESD is effective and safe for the treatment of difficult early esophageal cancer with submucosal fibrosis.

**Fig. 1:**
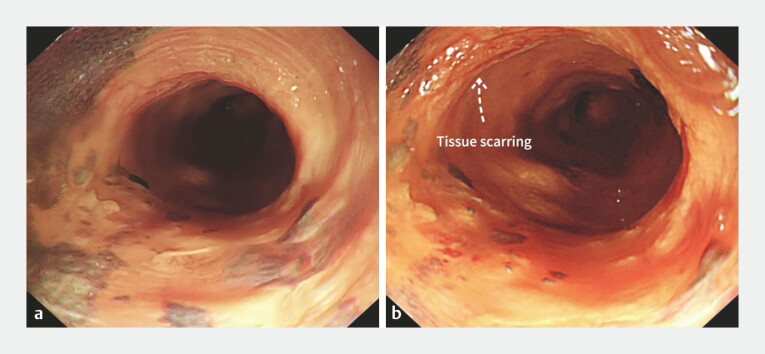
**a** Recurrent circumferential early esophageal cancer (24–35 cm from the incisors; tissue scarring). **b** Scar formation at the site 29–31 cm from the incisors after the previous ESD of the lesion. ESD, endoscopic submucosal dissection.

**Fig. 2:**
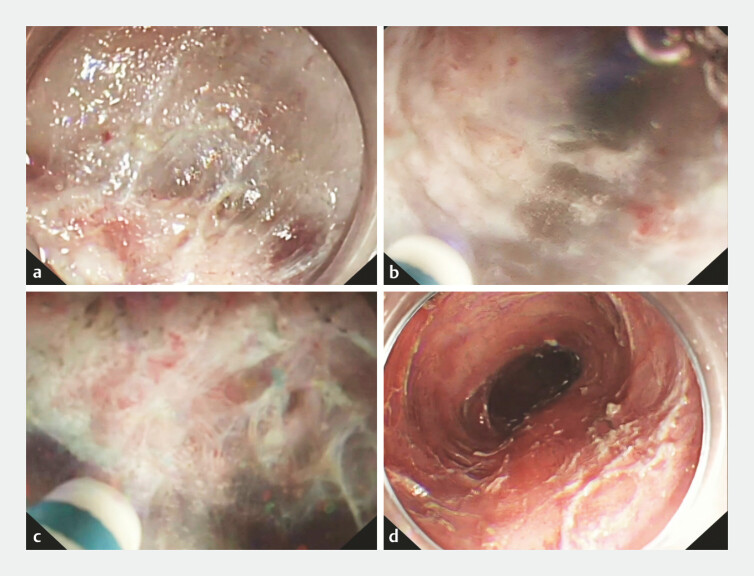
**a** Clip traction could not well expose the submucosa and the cutting line. **b** Under the action of the buoyancy of water, the cutting line in the submucosa was fully exposed. **c** The submucosal dissection was carried out smoothly. **d** After the operation, the wound was intact and smooth, and no adverse events such as muscular layer injury occurred.

**Fig. 3:**
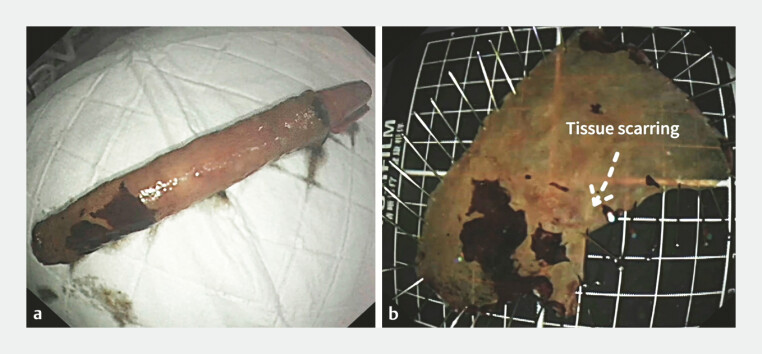
**a** The resected esophageal lesion in an en bloc fashion (tissue scarring). **b** The resected esophageal lesion in an en bloc fashion (tissue scarring).

Underwater ESD for resecting circumferential early-stage esophageal cancer recurring at the scar of the previous ESD site. ESD, endoscopic submucosal dissection.Video 1

Our experience indicates that underwater ESD can not only make the dissection of lesions at the scar site faster but also avoid damaging the muscularis propria or even causing perforation during the submucosal dissection process.

Endoscopy_UCTN_Code_TTT_1AO_2AG_3AD

## Notes

Endoscopy E-Videos https://eref.thieme.de/e-videos
*E-Videos* is an open access online section of the journal *Endoscopy* , reporting on interesting cases and new techniques in gastroenterological endoscopy. All papers include a high-quality video and are published with a Creative Commons CC-BY license. Endoscopy E-Videos qualify for HINARI discounts and waivers and eligibility is automatically checked during the submission process. We grant 100% waivers to articles whose corresponding authors are based in Group A countries and 50% waivers to those who are based in Group B countries as classified by Research4Life (see: https://www.research4life.org/access/eligibility/ ). This section has its own submission website at https://mc.manuscriptcentral.com/e-videos .
